# Mitochondria - metabolism and beyond

**DOI:** 10.1186/1741-7007-12-37

**Published:** 2014-05-27

**Authors:** Miranda Robertson

**Affiliations:** 1

## 

The past twenty years or so have seen a revolution in the perception of mitochondria, from the discrete bioenergetic organelles of 20^th^-century textbooks to a network undergoing constant fission and fusion regulated by cellular needs and physiological state, engaging with other membrane systems, and signalling to the machinery of the cytoplasm the metabolic status of the cell.

**Figure 1:**
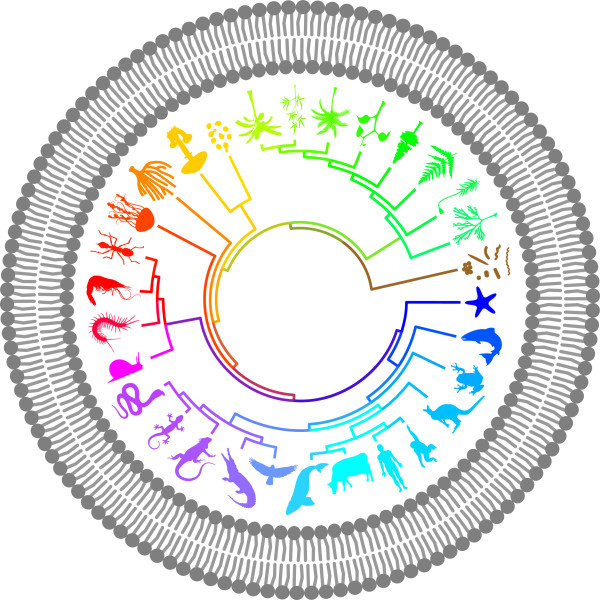
**The *BMC Biology *** **iconic image, in which a rootless phylogenetic tree is enclosed in a protocellular lipid bilayer.**

In parallel with the evolution of thinking about the cell biology of mitochondria has come a resurgence of interest in the classical biochemistry of metabolism, inspiring the headline in an article in *Science* not long ago, ‘Metabolism is not boring’ [1] – a revelation consequent in large part on the rediscovery of the Warburg effect and the realization that the metabolic demands on cancer cells are a point of vulnerability with the potential for new approaches to their destruction.

These developments, with the old mysteries of mitochondrial origins as endosymbionts, the complexities of their inheritance and bizarre dvision of genetic labor with the nucleus, and the breathtaking biophysical elegance of the electron transport chain, would together be more than enough to make mitochondria an irresistible topic for a collection of articles reflecting both the achievements and the emerging trends in recent research; and these are by no means all that is making mitochondria a focus of still-increasing topical interest.

Hence the collection that we launch this month with two reviews providing overviews of two of the most prominent cell biological topics in research on mitochondria.

Laura Lackner, who has helped establish some critical characteristics and functions of the working connection between mitochondria and the endoplasmic reticulum, writes on the dynamics of the mitochondrial network [2], how its continual fusion and fission are controlled, the functional causes and consequences of these dynamics, and what is known of their underlying mechanisms. Navdeep Chandel, whose principal research interest is in reactive oxygen species signalling in cancer cells, writes on the emergence and current status of mitochondria as signalling organelles, from the original recognition of their role in activating apoptosis to the part they play in modulating cellular metabolism in response to extra demands or stresses [3].

The extra demands and stresses that operate on mitochondria in cancer cells and in physiological extremes such as starvation, hypoxia, or endurance exercise, and the role of mitochondria in disease and aging, are the special concerns of our sister journals *Cancer & Metabolism*, *Extreme Physiology & Medicine*, and *Longevity & Healthspan*, which join us in putting together the series for which the articles by Lackner and Chandel are the inaugural contributions for *BMC Biology*.

It will be clear from these articles how much is still to be learned about how the machinery of mitochondria engages with that of the cell – a relationship that must have involved considerable evolutionary additions and adjustments on both the cellular and the mitochondrial side since the (presumed) engulfment of the original bacterial endosymbiont. Moreover mitochondria vary substantially from one tissue to another, with adaptations to the special metabolic needs of different tissues (see for example [4]), and across phylogeny – reflecting perhaps the neo-Lamarckian capture of functional plasticity in genomic changes to allow the evolution of functional diversity [5].

With the help of Guest Editors Jodi Nunnari and Peter Walter we are aiming to cover as many as we can of the absorbing topics that fall within the biology of mitochondria, though it would probably be rash to promise to include all of them. But we shall be welcoming submissions on any of the many issues still to be resolved in the origins, evolution, cell biology, metabolic adaptations and disease relationships of these extraordinary organelles – up to any including what they can tell us about the migratory paths of our prehistoric ancestry.
